# The safety and feasibility of laparoscopic redo surgery for recurrent Crohn’s disease: A comparative clinical study of over 100 consecutive patients

**DOI:** 10.1002/ags3.12534

**Published:** 2021-12-16

**Authors:** Takayuki Ogino, Yuki Sekido, Tsuyoshi Hata, Norikatsu Miyoshi, Hidekazu Takahashi, Mamoru Uemura, Hirofumi Yamamoto, Yuichiro Doki, Hidetoshi Eguchi, Tsunekazu Mizushima

**Affiliations:** ^1^ Department of Gastroenterological Surgery Osaka University Graduate School of Medicine Suita Japan; ^2^ Department of Therapeutics for Inflammatory Bowel Diseases Osaka University Graduate School of Medicine Suita Japan

**Keywords:** adhesion, Crohn's disease, laparoscopic surgery, redo surgery

## Abstract

**Background:**

Despite advances in medical treatments, most patients with Crohn's disease (CD) will still require surgery, with 20%‐50% needing redo surgery within 10 years after the primary procedure. There is no consensus on the application of laparoscopic redo surgery for recurrent CD.

**Methods:**

This study included 107 patients with CD who underwent surgery from 2012 to 2020 at Osaka University Hospital. All procedures were laparoscopic. Patients were grouped based on whether the surgery was redo or primary for evaluation of the safety and feasibility of laparoscopic redo surgery.

**Results:**

The study included 40 patients undergoing redo surgery and 67 having primary surgery. The median age at the time of the procedure was higher for those undergoing redo surgery (43 years vs 34 years, *P* < 0.0031), as were the duration of CD (16.5 years vs 8.3 years, *P* < 0.0012) and number of operating minutes (231.0 min vs 169.0 min, *P* < 0.0001). The remnant bowel length was shorter in the redo surgery group (270.0 cm vs 410.0 cm, *P* < 0.0001). Rates of open conversion were comparable between the two groups (10.0% vs 3.0%, *P* = 0.127), as were postoperative complications (32.5% vs 20.9%, *P* = 0.1812).

**Conclusions:**

These results suggest that laparoscopic redo surgery is safe and feasible, with comparable conversion rates and postoperative complications in experienced institutions.

## Graphical abstract

There is no consensus on the application of redo surgery for recurrent CD. Here, we evaluated the safety and feasibility of laparoscopic redo surgery.

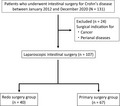

## 1 INTRODUCTION

Surgery plays a large role in the management of Crohn's disease (CD). With advances involving various biologics and immunomodulators, control of intestinal inflammation and treatment outcomes have been greatly improved.^1^ However, 70%‐80% of CD patients require surgery during the disease course, and some are at risk for needing redo procedures.^2^ Recent studies report overall cumulative rates of redo surgery at 20%‐50% within 10 years after the primary surgery.^3^, ^4^ Age, preoperative smoking, perianal disease, disease location in the ileocolon, perforating disease, positive microscopic resection margin, and steroid use have been reported as risk factors for redo surgery.^3^, ^4^

No other intestinal disease requires redo surgery as often as CD does. Most CD patients who need redo surgery experience complications such as abscesses, strictures, and intestinal fistulas.^3^ They also have an increased risk for postoperative complications, such as intra‐abdominal sepsis and anastomotic leakage.^5^, ^6^, ^7^ Results of previous studies indicate that patients with a history of abdominal surgery for CD should be excluded from laparoscopic surgery.^8^ Despite the high need for redo surgery, conclusive evaluations of the feasibility and safety of a laparoscopic approach are lacking. Meanwhile, CD surgeons are expected not only to focus on the current surgery but also to consider setting the stage as optimally as possible for a likely redo surgery.

A primary surgery for the non‐penetrating type of ileocecal lesion has been widely accepted as a good indication for laparoscopic surgery. Several studies have demonstrated the benefits of laparoscopic surgery for simple CD.^9^, ^10^ However, laparoscopic surgery for complex CD is associated with technical difficulties because of features such as widespread inflammation, mesenteric thickness, abscess, and fistula. In particular, redo surgery has often involved dealing with a dense and strong adhesion, making the procedure much more difficult. Thus, few reports are available describing laparoscopic redo surgery for recurrent CD, and of available findings, specific technical issues have been resolved, but the safety, feasibility, and utility have not.

In our experienced institution, we have been aggressively applying laparoscopic surgery in this patient group to minimize abdominal wall destruction and reduce postoperative inflammation. In this study, we assessed the outcomes of laparoscopic redo surgery for recurrent CD compared to outcomes with primary surgery.

## 2 METHODS

### 2.1 Patients

Since 2012, all procedures in our institution have involved a laparoscopic approach, whether primary or redo surgery. Single incisional laparoscopic surgery (SILS) was selected in principle. Additional ports were used when multiple organ resection was required, and use of more than two additional ports was defined as MULTI. Hand‐assisted laparoscopic surgery (HALS) was selected when extensive colectomy was required. HALS was indicated for patients with extensive colonic lesions, whereas SILS was indicated for patients with ileocolic lesions, ileocolic anastomotic lesions, or small intestinal lesions. Open conversion was considered depending on the intraoperative situation. The skin incisions used for each approach are shown in Figure S1.

**FIGURE 1:**
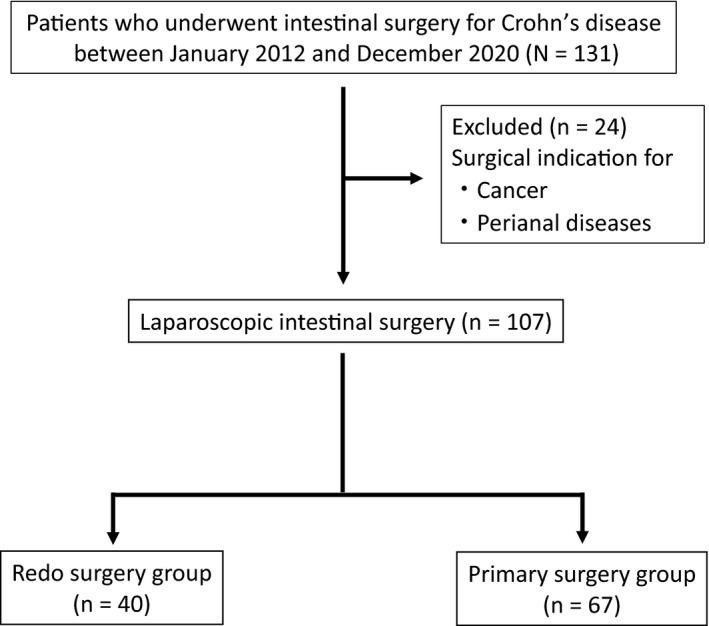
Flowchart of patient selection

From January 2012 to December 2020, a total of 131 consecutive patients who underwent surgery for CD intestinal lesions in Osaka University Hospital were included in this study. Patients with surgical indications for perianal diseases or cancer were excluded (Figure 1). The patients were grouped by surgery type (primary or redo), and laparoscopic surgery outcomes were evaluated between the two groups.

Indications for the surgeries were determined in inflammatory bowel disease (IBD) treatment team conferences attended by gastroenterologists, colorectal surgeons, radiologists, nutritionists, and nurses. All surgeries were performed by two qualified and board‐certified colorectal surgeons with established endoscopic surgical skills.

### 2.2 Surgical technique for redo surgery

All surgery was performed under general anesthesia, with the patient placed in the lithotomy position. An initial laparotomy (3‐4 cm) was made in the umbilicus, and adhesions around the previous wound were dissected. For the redo surgeries, most of the surgical sites were located in the ileocolic anastomosis. In these cases, a SILS device with one camera port and two manipulation ports was fitted for performing intra‐abdominal procedures, as previously described.^11^ After pneumoperitoneum was established, another port was added at the right lower site (planned site of drainage tube) if necessary.

Laparoscopic redo surgery involved several key features. Sharp and cold dissection was performed for adhesiolysis to minimize the risk of intestinal injury, along with careful manipulation of the intestine and mesentery without grasping inflamed, thickened, and edematous sites. Fistulas between the intestine, abdominal wall, urinary bladder, and inflamed lesion were divided intracorporeally using a stapler if necessary. A cranial approach often allowed easy identification of the retroperitoneal organs. Mobilization of the hepatic flexure and small bowel near the terminal ileum was performed first, and the recurrent area then was approached. After complete mobilization, the affected intestines were exteriorized, and resection and anastomosis were performed extracorporeally. An extension of the skin incision was sometimes needed in cases of bulky mass with inflammation. Careful exploration of the large and small intestines was performed, and the presence or absence of obstruction was confirmed by passage of a balloon inflated to 2 cm from the terminal ileum to the Treitz ligament. After opening of the intestinal lumen at the planned resection line, the inflated balloon was inserted from the site. We folded the intestinal tract like an accordion and forwarded the balloon to the Treitz ligament to assess for the presence or absence of obstruction. For dividing thickened mesentery, we found that ultrasonically activated devices were not sufficient to seal the large blood vessels, so we added transfixion sutures. The affected segment was resected, and a functional end‐to‐end anastomosis was performed. The stapled lines were oversewn and the defects of the mesentery closed routinely. In cases of a short affected lesion, strictureplasty was performed using the hand‐sewn technique. An active drainage tube was routinely placed in Morrison's pouch or Douglas’ pouch as previously described.^12^

### 2.3 Data collection

Data were retrospectively collected for the comparison between CD patients with redo surgery and those with primary surgery. Patient characteristics included age, sex, body mass index, the American Society of Anesthesiologists physical status (ASA‐PS), age at diagnosis, duration of disease, location, prognostic nutrition index, preoperative data, surgical indications, preoperative medication, loss of response, and surgical history. Perioperative data included approach, surgical procedure, operative time, blood loss, length of skin incision, conversion to open surgery, remnant bowel length, postoperative complications, excessive perioperative inflammation, and length of hospital stay.

Loss of response was defined as increased doses of biologics, shortened dosing interval, or changed other biologics within 6 months before surgery. Postoperative complications were defined as complications occurring within 30 days after surgery. Excessive perioperative inflammation was defined as preoperative C‐reactive protein (CRP) ≥0.4 mg/dL combined with CRP ≥3.0 mg/dL at postoperative day 7 and/or postoperative peak CRP ≥10 mg/dL.

### 2.4 Statistical analysis

All statistical analyses were conducted using GraphPad Prism software, version 5.0 b. The chi‐square test and Fisher's exact test were used to compare and analyze categorical variables. All analyses were two‐tailed with *P* < 0.05 considered significant.

### 2.5 Ethics statement

The protocol for this study was approved by the institutional review board of Osaka University Graduate School of Medicine (#15028). The procedures conformed to the provisions of the Declaration of Helsinki. Written informed consent was obtained from all patients for use of their clinical data.

## 3 RESULTS

### 3.1 Patient characteristics

In our institution, all intestinal surgeries for CD were started using a laparoscopic approach. A total of 131 consecutive patients underwent intestinal surgery for CD during the study period. We excluded 24 patients with surgical indication for cancer or perianal disease, so that data for 107 patients were analyzed in this study (Figure 1). Of these, 40 (37.4%) had redo surgery and 67 (62.6%) had no history of abdominal surgery for CD.

Patient characteristics are shown in Table 1. The median age at operation was 43 years in the redo group, which was older than patients undergoing primary procedures, who had a median age of 34 years (*P* < 0.0031). The two groups were comparable for sex, body mass index, proportion of ASA‐PS, and median age at CD diagnosis. Duration of CD was 16.5 years for those undergoing redo surgery, significantly longer than in the primary group (8.3 years; *P* < 0.0012). The two groups did not differ regarding disease location, perianal disease, preoperative prognostic nutrition index, or CRP values. Preoperative treatments were comparable with regard to anti‐tumor necrosis factor‐α, anti‐interleukin 12/23p40, and corticosteroid therapy. Patients undergoing redo procedures had a numerically higher frequency of immunomodulator use, but not significantly so (*P* = 0.052). Loss of response, rates of emergency operation, and surgical indication were also comparable between the two groups.

**TABLE 1:** Patient characteristics

	Redo (n = 40)	Primary (n = 67)	*P*
Age at operation (y), median ± SD	43.0 ± 11.9	34.0 ± 12.4	0.0031
Sex, n (%)			0.2015
Male	32 (80.0)	46 (68.7)	
Female	8 (20.0)	21 (31.3)	
Body mass index (kg/m^2^), median ± SD	18.9 ± 3.1	19.0 ± 3.0	0.9792
ASA‐PS, n (%)			0.3947
1	13 (32.5)	25 (37.3)	
2	26 (65.0)	42 (62.7)	
3	1 (2.5)	0 (0.0)	
Age at diagnosis (y), median ± SD	26.5 ± 9.6	25.0 ± 11.6	0.5859
Duration of disease (y), median ± SD	16.5 ± 11.7	8.3 ± 8.0	0.0012
Locations, n (%)			0.6625
Small bowel	18 (45.0)	32 (47.8)	
Colon	3 (7.5)	8 (11.9)	
Ileocolon	19 (47.5)	27 (40.3)	
Perianal diseases	27 (67.5)	41 (61.2)	0.512
Preoperative prognostic nutrition index, median ± SD	42.8 ± 10.8	40.4 ± 8.6	0.1125
Preoperative CRP (mg/dL), median ± SD	0.16 ± 2.21	0.46 ± 3.53	0.2127
Preoperative medication, n (%)^a^			
Anti‐tumor necrosis‐α	24 (60.0)	38 (56.7)	0.7392
Anti‐interleukin 12/23p40	5 (12.5)	8 (11.9)	0.9317
Steroid	2 (5.0)	10 (14.9)	0.1154
Immunomodulator	16 (40.0)	15 (22.4)	0.052
Loss of response	19 (47.5)	30 (44.8)	0.7844
Emergency operation, n (%)	1 (2.5)	7 (10.4)	0.1305
Surgical indication, n (%)			0.091
Fistula/abscess	15 (37.5)	34 (50.7)	
Stenosis	19 (47.5)	32 (47.8)	
Bleeding	1 (2.5)	1 (1.5)	
Ileostomy closure	5 (12.5)	0 (0.0)	
Duration from previous surgery (y), median ± SD	9.9 ± 8.5	‐	
Surgical history of bowel resection for CD, n (%)			
1	23 (57.5)	‐	
2	14 (35.0)	‐	
≥3	3 (7.5)	‐	
Approach in previous surgery, n (%)			
Open	33 (82.5)	‐	
Laparoscopic	7 (17.5)	‐	

^a^ Including duplicates.

For patients having redo procedures, the time since the previous surgery was 9.9 years. In this group, 23 patients (57.5%) had undergone one surgical bowel resection, 14 (35.0%) had a history of two surgeries, and three (7.5%) had undergone more than three surgeries. The approach used for previous surgeries was open in 33 patients (82.5%) and laparoscopic in seven patients (17.5%).

### 3.2 Surgery‐related factors

The proportions of the different laparoscopic surgical approaches were comparable between the two groups (*P* = 0.5895). In the redo group, 82.5% had SILS, 5.0% had MULTI, and 12.5% had HALS procedures. In the primary surgery group, 77.6% had SILS, 3.0% had MULTI, and 19.4% had HALS (Table 2). In the redo group, 62.5% had ileocecal resection or ileocolectomy, compared with 53.7% undergoing primary surgery. For some patients in both groups, additional procedures were performed, including partial resection of the small intestine (35.0% of redo and 34.3% of primary surgery patients), right hemicolectomy (2.5% of redo and 3.0% of primary surgery patients), left hemicolectomy (5.0% redo, 4.5% primary), subtotal colectomy (10.0% redo, 14.9% primary), strictureplasty (17.5% redo, 14.9% primary), stoma creation (17.5% redo, 7.5% primary), and stoma closure (10.0% in redo). Operating time was 231.0 ± 93.9 min for the redo group and 169.0 ± 65.1 min for the primary surgery group. As expected, operating time was significantly longer with redo surgeries, given that adhesiolysis was often necessary (*P *< 0.0001). Compared with the primary surgery group, the redo group also had more operative blood loss (*P* = 0.0372), longer skin incisions (*P* = 0.0272), and shorter remnant bowel length (*P* < 0.0001). An incisional extension was performed in 13 patients (32.5%) undergoing redo and 17 (25.4%) having a primary procedure. Of these, open conversion was performed in four patients in the redo group and two in the primary group. The additional length was 2.0 ± 2.8 cm for the redo patients and 1.7 ± 2.0 cm for the primary group. Rates of conversion to open surgery were not significantly different between the two groups (*P* = 0.127). There were four cases of open conversion in the redo group. In the first case, inflammation had spread to the retroperitoneum, and the boundary with the left ureter was unclear. It was difficult to confirm the left ureter in the second case, and there was a severe adhesion between the jejunum and the transverse colon in the third case. The fourth case also involved a strong adhesion between the ileum and the ileo‐rectal anastomosis. The primary group included two conversions, one with marked intestinal dilatation and intestinal injury and the other with a widespread enterocutaneous fistula associated with difficult mobilization from the abdominal wall, ileum, and ileal mesentery.

**TABLE 2:** Operative findings

	Redo (n = 40)	Primary (n = 67)	*P*
Approach, n (%)			0.5895
SILS	33 (82.5)	52 (77.6)	
MULTI	2 (5.0)	2 (3.0)	
HALS	5 (12.5)	13 (19.4)	
Surgical procedure, n (%)^a^			
Ileocecal resection/ileocolectomy	25 (62.5)	36 (53.7)	
Partial resection of small intestine	14 (35.0)	23 (34.3)	
Right hemicolectomy	1 (2.5)	2 (3.0)	
Left hemicolectomy	2 (5.0)	3 (4.5)	
Subtotal colectomy	4 (10.0)	10 (14.9)	
Strictureplasty	7 (17.5)	10 (14.9)	
Stoma creation	7 (17.5)	5 (7.5)	
Stoma closure	4 (10.0)	0 (0.0)	
Operating time (min), median ± SD	231.0 ± 93.9	169.0 ± 65.1	<0.0001
Blood loss (mL), median ± SD	185.0 ± 188.1	100.0 ± 330.8	0.0372
Skin incision (cm), median ± SD	5.0 ± 2.7	4.0 ± 1.5	0.0272
Conversion to open surgery, n (%)	4 (10.0)	2 (3.0)	0.127
Remnant bowel length (cm), median ± SD	270.0 ± 99.4	410.0 ± 94.6	<0.0001

^a^ Including duplicates.

### 3.3 Short‐term outcomes

Short‐term outcomes are shown in Table 3. Postoperative complications were comparable between the two groups (32.5% redo vs 20.9% primary; *P* = 0.1812). Wound infection occurred in eight patients in the redo group and five having primary surgery (*P* = 0.0698). No redo patients had anastomotic leakage, but one primary surgery patient did (*P* = 1.000). Three in each group had small bowel obstruction (*P* = 0.6692), and three redo patients and one primary surgery patient had an intra‐abdominal abscess (*P* = 0.1460). One redo patient and three primary surgery patients had gastrointestinal bleeding, and two primary surgery patients had intra‐abdominal bleeding (*P* = 0.4073). No redo patients had a catheter infection, but three primary surgery patients did (*P* = 0.2911). The two groups did not differ in postoperative day 7 CRP or postoperative peak CRP. Excessive perioperative inflammation, which is associated with recurrence,^13^ also was comparable between the two groups. The groups also did not differ in length of postoperative hospital stay (*P* = 0.8653), and there were no reoperations or postoperative mortalities.

**TABLE 3:** Short‐term outcomes

	Redo (n = 40)	Primary (n = 67)	*P*
Complications, n (%)^a^	13 (32.5)	14 (20.9)	0.1812
Wound infection	8 (20.0)	5 (7.5)	0.0698
Anastomotic leakage	0 (0.0)	1 (1.5)	1.000
Small bowel obstruction	3 (7.5)	3 (4.5)	0.6692
Intra‐abdominal abscess	3 (7.5)	1 (1.5)	0.146
Bleeding	1 (2.5)	5 (7.5)	0.4073
Catheter infection	0 (0.0)	3 (4.5)	0.2911
Post‐operative day 7 CRP (mg/dL), mean ± SD	1.60 ± 4.43	1.61 ± 3.80	0.2527
Postoperative peak CRP (mg/dL), mean ± SD	9.01 ± 5.65	7.06 ± 6.92	0.4654
Excessive perioperative inflammation, n (%)	11 (27.5)	18 (26.9)	0.9431
Reoperation within post‐operative day 30	0 (0.0)	0 (0.0)	
Mortality, n (%)	0 (0.0)	0 (0.0)	
Postoperative hospital stay (days), median ± SD	24.0 ± 11.5	25.0 ± 9.3	0.8653

^a^ Including duplicates.

## 4 DISCUSSION

Laparoscopic colorectal cancer surgery has become widespread and has been rapidly accepted.^14^ In IBD, a laparoscopic approach has already been accepted for simple CD cases, with fewer complications and improved early postoperative outcomes.^9^, ^10^ The reported benefits of laparoscopic surgery include reduced postoperative pain, improved respiratory function, less wound infection, less incisional hernia, less small bowel obstruction, earlier resumption of bowel function, shorter hospital stay, and better cosmesis.^9^, ^10^, ^15^, ^16^, ^17^ Especially, reduced rates of small bowel obstruction would indicate lower postoperative adhesion,^18^ and suggest attractive advantages in consideration of a later redo surgery (see Supporting Information for case descriptions).

Several studies have described a laparoscopic approach for recurrent CD. Most results indicate a longer operation time, but without significant differences in bleeding, open conversion rates, or complication rates compared with primary surgery.^19^, ^20^, ^21^ Our study also suggests that laparoscopic redo surgery requires a longer operative time, but still with comparable open conversion and postoperative complication rates compared with primary surgery. Studies comparing laparoscopic and open redo surgery have found comparable operation time and complication rates between the two,^17^, ^22^, ^23^ but with the advantages of a shorter skin incision, less wound infection, and faster postoperative recovery with laparoscopy.^24^ The open conversion rates in these studies were 11% to 31%, and risk factors were dense adhesions, bulky mass, fistula formation, and intraoperative intestinal injury.^21^, ^22^, ^24^, ^25^, ^26^, ^27^ However, careful interpretations of previous findings are necessary because of patient selection bias for laparoscopic redo surgery in these studies. Our current work represents the first study of consecutive patients all undergoing laparoscopic surgery, regardless of primary or redo surgery, avoiding this selection bias.

Consistent with previous results, we found that laparoscopic redo surgery took longer but did not increase postoperative complications. As noted, laparoscopic redo surgery often entails high technical difficulty because of massive adhesions, the fragility of inflamed lesions, thickened mesentery, inflammatory masses with abscess, and fistulas. In this study, adhesions to the abdominal wall at the previous surgical site could be dissected in all cases. For exteriorizing affected intestines, a midline skin incision was beneficial for evaluation of the entire small intestine and the need for open conversion. Most reasons for open conversion were intraoperative decisions about injury risk to other organs because their orientation could not be established during surgery. To prevent such situations arising during a redo surgery, we recommend two techniques, especially in cases of partial ileal resection or ileocecal resection as a primary surgery. One is mobilization of the right colon to the minimum necessary, and the second is preservation of the prepancreatic fascia. These points could lead to significant advantages in a later redo surgery. We also consider SILS a promising technique that could avoid unnecessary intestinal injury, minimize the skin incision, and establish additional ports in the best positions.^28^

The present study has several limitations. Its design was retrospective, and it was conducted in a single institution specializing in IBD and laparoscopic surgery. Most surgeries were performed by two board‐certified surgeons, so the findings cannot be generalized to inexperienced centers and surgeons. Perioperative medication and diet, which would affect CD surgery, were selected largely according to clinician discretion and patient condition. This study also identified postoperative hospital stays as long as 20 days or more, whereas most previous results involve hospital stays of less than 1 week, whether for primary or redo surgery.^7^, ^16^, ^20^, ^23^, ^26^ Finally, patients were allowed to have an elementary diet after negative CRP in accordance with institutional therapeutic guidelines based on previous results that postoperative inflammation is involved in CD recurrence.^12^, ^25^, ^29^

With the increase in CD patients, laparoscopic redo surgery for recurrent CD will become a common procedure in the near future. Our results suggest that laparoscopic redo surgery is safe and feasible in experienced institutions. Although the quantification of adhesion severity is quite difficult, we have found that the adhesions to the abdominal wall were not very strong in case of previous surgery performed laparoscopically (see Supporting Information for case descriptions). Fewer adhesions would set the stage for easier surgery if a redo is required.

## DISCLOSURE

Conflict of Interest:The authors declare no conflicts of interest for this article.

## Supporting information

Figure S1

Supplementary Material
