# Supplementary figures and images for: The safety and feasibility of laparoscopic redo surgery for recurrent Crohn’s disease: A comparative clinical study of over 100 consecutive patients
Source: Ann Gastroenterol Surg. 2021 Dec 16;6(3):405–11. doi: 10.1002/ags3.12534 (PMC9130919; doi:10.1002/ags3.12534)

## Slide 1
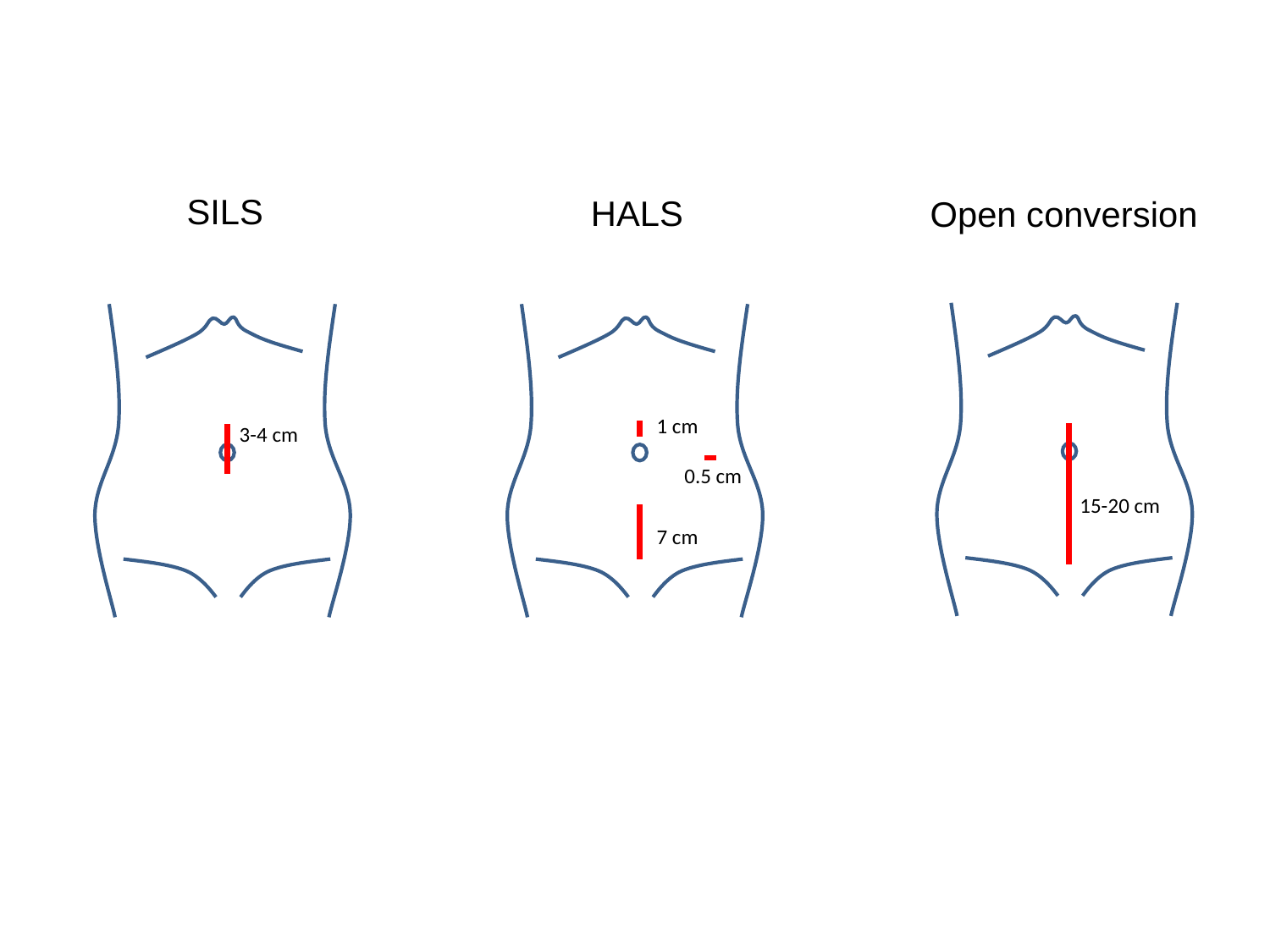

SILS
HALS
Open conversion
1 cm
3-4 cm
0.5 cm
15-20 cm
7 cm

Supplement: Supplementary file 1 — Figure S1 [file AGS3-6-405-s001.pptx]
